# Complete blood counts as potential risk factors of early dissemination to liver and lungs in resected colorectal cancer: a retrospective cohort study

**DOI:** 10.1007/s00384-024-04802-9

**Published:** 2025-01-21

**Authors:** Marta Popęda, Jolanta Żok, Bartłomiej Tomasik, Renata Duchnowska, Michał Bieńkowski

**Affiliations:** 1 Department of Pathomorphology, Medical University of Gdańsk, Gdańsk, Poland; 2 Department of Oncology, Military Institute of Medicine, Warsaw, Poland; 3 Department of Oncology and Radiotherapy, Medical University of Gdańsk, Gdańsk, Poland

**Keywords:** Colorectal cancer, Liver metastasis, Lung metastasis, Complete blood counts, Risk assessment

## Abstract

**Purpose:**

Liver and lung metastases demonstrate distinct biological, particularly immunological, characteristics. We investigated whether preoperative complete blood count (CBC) parameters, which may reflect the immune system condition, predict early dissemination to the liver and lungs in colorectal cancer (CRC).

**Methods:**

In this retrospective single-centre study, we included 268 resected CRC cases with complete 2-year follow-up and analysed preoperative CBC for association with early liver or lung metastasis development. Next, selected CBC and clinicopathological parameters were analysed with uni- and multivariable Cox regression. Independent factors affecting liver or lung metastasis-free survival were incorporated into composite scores, which were further evaluated with receiver operating characteristic (ROC) curves and dichotomised using a modified, specificity-focused, Youden approach to identify particularly high-risk patients.

**Results:**

Compared to metastasis-free patients, early liver metastases were related to decreases in red blood cells, haematocrit, lymphocytes and elevated monocyte-to-lymphocyte ratio, while lung metastases to lower eosinophil counts. A composite score of independent factors (erythrocytopenia, lower lymphocyte count and pN) yielded HR of 8.01 (95% CI 3.45–18.57, *p* < 0.001) for liver-specific metastasis-free survival (MFS). For lung-specific MFS, the combination of eosinopenia, pN and primary tumour location showed HR of 13.69 (95% CI 4.34–43.20, *p* < 0.001).

**Conclusion:**

Early CRC metastases to the liver and lungs are associated with partially divergent clinicopathological and peripheral blood features. We propose simple, clinically implementable scores, based on routinely assessed parameters, to identify patients with an increased risk of early dissemination to the liver or lungs. After validation in independent cohorts, these scores may provide easily available prognostic information.

**Supplementary Information:**

The online version contains supplementary material available at 10.1007/s00384-024-04802-9.

## Introduction

Colorectal cancer (CRC) is the third most common cancer and the second cause of cancer-related mortality (accounting for nearly 10% of cases) [1]. The majority of CRC-related deaths are linked with metastatic dissemination, which occurs in a third of patients with local or regional disease [2, 3]. Importantly, CRC is characterised by early recurrence, with 65% of relapses occurring within the first 2 years after resection, and over 90% within the first 5 years [4, 5]. Thus, efficient monitoring during the first years of follow-up is crucial [6].

The optimal management of nonmetastatic CRC remains controversial. The systemic adjuvant treatment is generally recommended for stage III tumours, and yet about 30% of cases recur within 5 years [4, 7–10]. Despite that, the de-escalation of therapy has recently been postulated [11]. In turn, the recurrence rate is only slightly lower in earlier-stage cancer (about 18% in stage II and 12% in stage I), where systemic treatment is optional or not recommended [7–10]. This suggests that a significant proportion of patients is potentially subjected to over- or undertreatment, highlighting the need for better tools guiding clinical decisions. Mismatch repair status and Immunoscore, both reflecting immunogenicity of the tumour, are well-documented prognostic markers, yet not predictive for the standard systemic adjuvant treatment [12–14]. Several multi-gene assays and the measurement of post-surgical ctDNA levels have also been investigated [15–19] but have not entered patient management guidelines yet [7–10].

Complete blood counts (CBC) have recently gained substantial interest in this context as a non-invasive, routinely assessed source of clinically relevant information in CRC. For instance, platelets, known for a plethora of pro-metastatic properties in various cancers [20, 21], were reported to bear a prognostic value in both primary CRC and its liver metastases [22–24].

Metastasis of CRC demonstrates a distinct organotropism, with the liver being the most frequent distant site, followed by the lungs, affected in approximately 70% and 30% of patients with disseminated disease, respectively [25–27]. A growing body of evidence advocates for biological, in particular immunological, similarities between metastases to specific sites, regardless of their origin [28–31]. Metastases to the lungs tend to present higher immunogenicity [29], whereas those to the liver have a highly immunosuppressive microenvironment [28]. Accordingly, the CRC lung metastases typically show immune “hot” phenotype with an elevated proportion of CD4 + (Treg in particular) T cells and M2-like macrophages, while the immune “cold” liver metastases have reduced numbers of cancer-associated fibroblasts compared to the primary tumour (PT) or other metastatic sites [32]. The immune system plays a major role in shaping metastatic CRC [6], while the interactions between immune, stromal and tumour cells are involved in organ-specific metastases. Thus, we hypothesised that metastases to different organs may be reflected by different, specific changes in CBC, which may potentially be identified before the clinical manifestation of metastases.

Here, we investigated whether preoperative CBC parameters may carry prognostic information for the early development of liver and lung CRC metastases in addition to that of the clinicopathologic characteristics.

## Methods

### Patients

We did a retrospective analysis of consecutive patients with CRC who had undergone preoperative CBC measurement, PT resection and pathological diagnosis at the University Clinical Centre in Gdańsk, Poland, between May 2012 and June 2023. This study was approved by the Bioethical Committee of the Medical University of Gdańsk (NKBBN/688/2022) and performed under relevant guidelines and regulations, including the Helsinki Declaration of 1975 and STROBE recommendations [33]. Due to the retrospective character of the study, patient consent was not required.

**Fig. 1:**
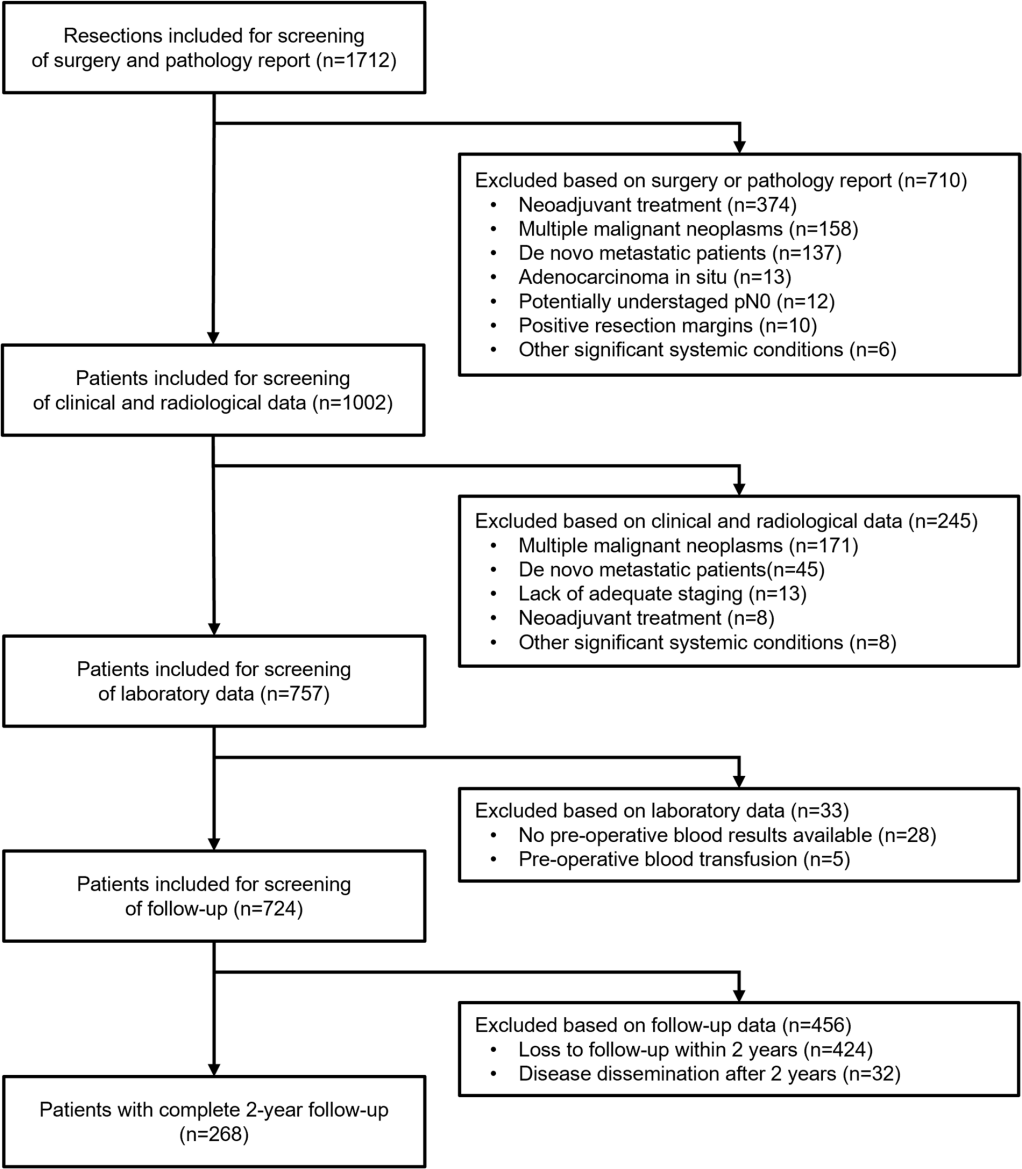
Flowchart of patient selection

A total of 1712 patients were screened for eligibility based on thorough curation of surgery and pathology reports, clinical, radiological and laboratory data. The inclusion criteria were (1) primary surgical resection of invasive colorectal cancer with clean margins, (2) adequate radiologic and pathologic staging, (3) availability of preoperative complete blood counts before the potential transfusion and (4) complete follow-up of at least 2 years or disease dissemination within 2 years; the exclusion criteria were (1) neoadjuvant treatment, (2) metastatic disease, (3) multiple malignant tumours, (4) carcinoma in situ, (5) other systemic conditions that significantly affect blood parameters and (6) metastatic dissemination after 2 years. A total of 268 patients fulfilled the criteria (Fig. 1).

### Complete blood counts

CBC were measured from routinely collected blood samples in the Central Clinical Laboratory of the University Clinical Centre in Gdańsk, Poland, following standard operating procedures. Preoperative CBC were defined as those performed within 30 days prior to primary CRC resection; if multiple measurements were available per patient, the one closest to the start of hospitalisation was included.

The extracted parameters included haemoglobin (HGB) and haematocrit (HCT) as well as red blood cell (RBC), platelet (PLT), neutrophil (NEUTR), lymphocyte (LYMPH), monocyte (MONO), eosinophil (EO) and basophil (BASO) counts. To account for the gender-specific differences in erythrocytes, all parameters were scaled according to the normal range (as presented in Table S1) with 0 as the middle of the normal range, while − 1 and 1 as the lower and upper margin, respectively. Selected parameters were also categorised according to normal range values. Platelet-to-lymphocyte ratio (PLR), neutrophil-to-lymphocyte ratio (NLR) and monocyte-to-lymphocyte ratio (MLR) were calculated based on the respective counts.

### Statistical analysis

Statistical analysis was performed using R statistical software (version 4.2.2 [34]). To decipher the CBC profile associated with organ-specific metastasis formation, patients who developed metastasis to the liver (*n* = 25) or the lungs (*n* = 12) were compared to those who remained metastasis-free (*n* = 206) during the follow-up. Association between continuous CBC parameters and histology or metastasis to the liver or lungs was evaluated using the Mann–Whitney–Wilcoxon test and further validated with 2-way ANOVA accounting for PT location. False discovery rate (FDR) correction was applied for multiple testing. Fisher’s exact test was used to evaluate the distribution of categorised clinicopathological variables and CBC levels. Metastasis-free survival was defined as the time from PT resection to the first evidence of metastases. Survival analysis was performed using Cox proportional hazard models, both univariable and multivariable, and visualised as Kaplan–Meier curves (*ggplot2* [35] and *survminer* [36] R packages). Simple models scoring the risk of liver or lung metastasis were established based on final multivariable Cox regression models, employing the Cox coefficients rounded to halves. The models were assessed using receiver operating characteristic (ROC, *pROC* package [37]) curves. Optimal thresholds were identified with a modification of Youden’s method with a double weight of specificity. Harrell’s C-index, calculated with the *cIndex* function from the *intsurv* package [38], was used to assess model performance. A *p*-value below 0.05 and FDR below 0.1 were considered statistically significant.

## Results

We included 268 CRC patients with complete 2-year follow-up in the study. The group comprised 20 mucinous and 248 adenocarcinomas not otherwise specified (NOS/tubular). The mucinous tumours were predominantly diagnosed in the right colon, in older patients, and presented a low metastasis rate (1/20, 5%) within 2 years post-resection (Table S2). Moreover, the discrepancies between CRC histologic subtypes manifested in preoperative CBC parameters, with mucinous tumour patients having lower RBC, HGB and HCT, while higher PLT counts (Table S3).

Thus, mucinous adenocarcinomas were excluded from further analysis. Out of the remaining 248 patients, 42 (17%) developed distant metastasis within 2 years post-resection, with a total number of 25 (10%) and 12 (5%) cases with liver and lung metastasis, respectively (Table S2; a detailed distribution of metastatic sites is provided in Figure S1). Isolated metastases accounted for 95% of liver and 42% of lung metastases. To ensure clinical utility, we did not differentiate between isolated and concurrent metastases to each organ. Instead, patients with any pattern of early dissemination to these organs were compared to those who remained metastasis-free during the 2-year follow-up period.

### Liver metastases

**Fig. 2:**
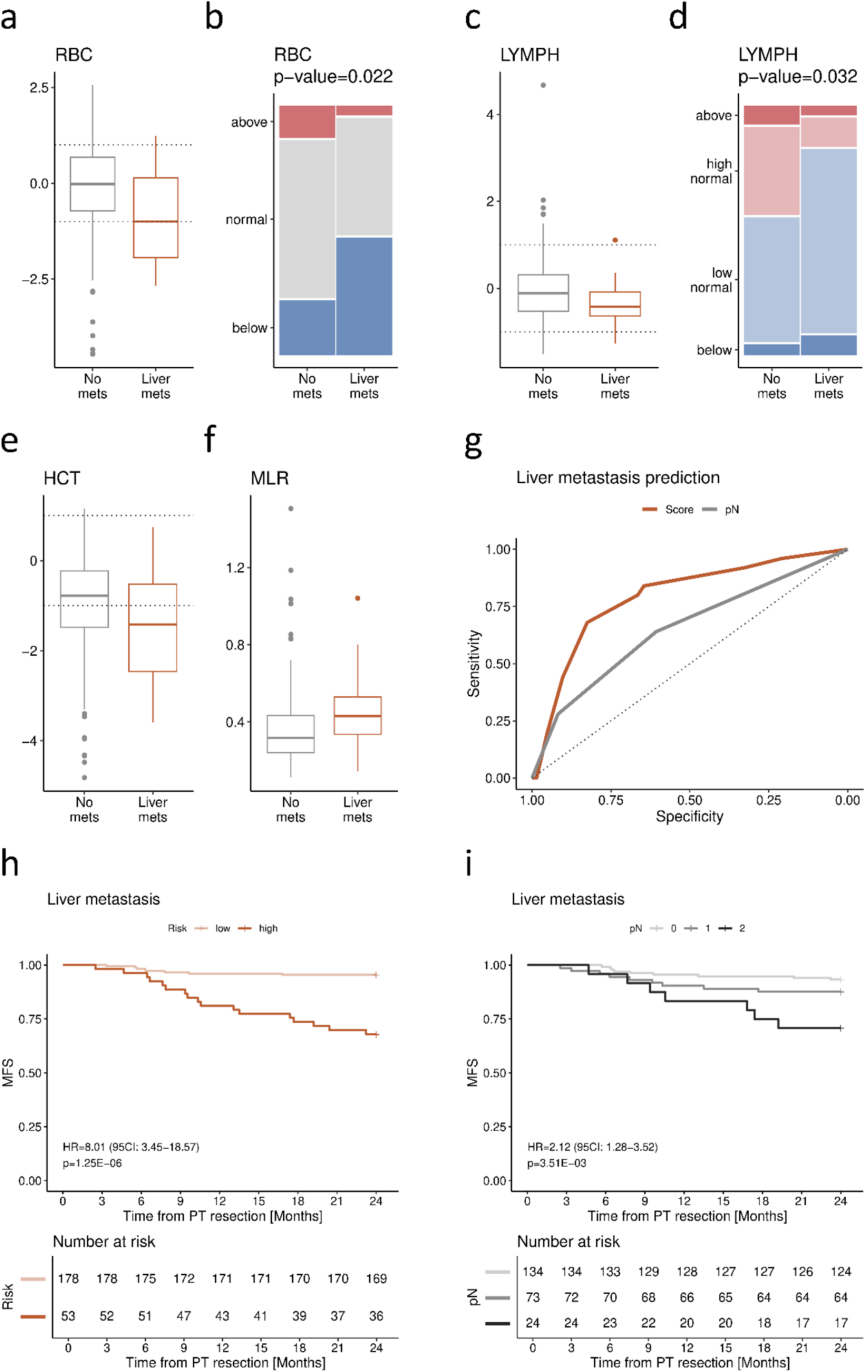
Features associated with liver metastasis. Distribution of RBC (**a**, **b**), LYMPH (**c**, **d**), HCT (**e**) and MLR (**f**) between patients who developed liver metastases (*n* = 25) vs. metastasis-free patients (*n* = 206). Continuous RBC (**a**), LYMPH (**c**) and HCT (**e**) are presented as scaled to the normal range, while MLR (**f**) as unscaled due to the lack of defined ranges. Categorisation performed based on the lower and upper margin of the normal range for RBC (**b**) or the lower margin, middle and upper margin of the normal range for LYMPH (**d**). Evaluated with the Mann–Whitney–Wilcoxon test and verified with 2-way ANOVA accounting for PT location for continuous variables (*p*-values and FDR presented in Table 2), or Fisher’s exact test for categorical variables. Receiver operating characteristic (ROC) curves for predicting dissemination to the liver based on the combined score (orange) and pN only (grey) (**g**). Kaplan–Meier curves for liver metastasis-free survival (MFS) for the combined score dichotomised according to the cut-off value selected with the specificity-focused Youden approach (**h**) as well as for pN only (**i**). Hazard ratio (HR) and the corresponding 95% confidence interval (CI) estimated with Cox proportional hazard model; pN analysed for the effect of a continuous increase (0/1/2)

Within 2 years post-resection, liver metastases were more prevalent in males (17/107, 16%; *p* = 0.032), patients with stage III disease (16/97, 16%; *p* = 0.007) and those with pN2 tumours (7/24, 29%; *p* = 0.006) in particular (Table 1). Patients who developed liver metastasis demonstrated lower RBC (FDR = 0.019; Fig. 2a) and LYMPH (FDR = 0.053; Fig. 2c) counts, HCT (FDR = 0.102; Fig. 2e) and elevated MLR (FDR = 0.019; Fig. 2f; all Mann–Whitney–Wilcoxon test) before primary tumour resection (Table 2). Of note, categorisation with reference-based cut-offs revealed significant differences in distribution among patients with no vs. liver metastasis for RBC (*p* = 0.022; Fig. 2b) and LYMPH (*p* = 0.032; Fig. 2d), further dichotomised by the lower margin and the middle of the normal range, respectively.

**Table 1:** Comparison of clinicopathological characteristics between patients developing early liver metastases and metastasis-free patients

Parameter		No mets (*n* = 206)		Liver mets (*n* = 25)		*p*-value
		*n*	%	*n*	%	
Age at diagnosis	Median (IQR)	66	59–73	69	62–76	2.16E-01
Gender	Female	116	56%	8	32%	**3.21E-02**
	Male	90	44%	17	68%	
Primary tumour location	Right colon	73	35%	6	24%	3.23E-01
	Left colon	103	50%	13	52%	
	Rectum	30	15%	6	24%	
pT	pT1	9	4%	0	0%	3.13E-01
	pT2	41	20%	2	8%	
	pT3	135	66%	19	76%	
	pT4	21	10%	4	16%	
pN	pN0	125	61%	9	36%	**6.08E-03**
	pN1	64	31%	9	36%	
	pN2	17	8%	7	28%	
Stage	I	42	20%	0	0%	**6.88E-03**
	II	83	40%	9	36%	
	III	81	39%	16	64%	

Patients who developed liver metastases within 2 years after resection vs. metastasis-free patients. Evaluated with Mann–Whitney–Wilcoxon test (age at diagnosis) and with Fisher’s exact test (other variables); significant comparisons (*p* < 0.05) are marked in bold

**Table 2:** Comparison of complete blood counts between patients developing early liver metastases and metastasis-free patients

Parameter		No mets	Liver mets	Liver mets vs. no mets		Liver metastasis		Location	
		Median	Median	*p*-value*	FDR*	*p*-value^#^	FDR^#^	*p*-value^#^	FDR^#^
Red blood cells (RBC)	Scaled	−0.02	−1.00	**3.19E-03**	**1.91E-02**	**6.05E-03**	**2.42E-02**	**1.58E-02**	**4.21E-02**
Haemoglobin (HGB)	Scaled	−0.80	−1.35	6.49E-02	1.30E-01	**3.62E-02**	**7.24E-02**	**3.88E-09**	**9.32E-08**
Haematocrit (HCT)	Scaled	−0.78	−1.42	**3.41E-02**	1.02E-01	**2.54E-02**	**5.54E-02**	**2.43E-08**	**2.92E-07**
Platelets (PLT)	Scaled	0.03	0.08	9.00E-01	9.47E-01	7.87E-01	8.21E-01	**7.19E-06**	**5.75E-05**
Neutrophils (NEUTR)	Scaled	0.02	−0.01	9.47E-01	9.47E-01	8.31E-01	8.31E-01	**3.50E-03**	**1.68E-02**
Lymphocytes (LYMPH)	Scaled	−0.11	−0.42	**1.31E-02**	**5.26E-02**	**2.41E-02**	**5.54E-02**	4.28E-01	5.37E-01
Monocytes (MONO)	Scaled	−0.01	0.18	1.45E-01	2.18E-01	4.77E-01	5.45E-01	2.25E-01	3.17E-01
Eosinophils (EO)	Scaled	−0.54	−0.67	4.06E-01	4.87E-01	4.48E-01	5.37E-01	1.91E-01	2.87E-01
Basophils (BASO)	Scaled	−0.40	−0.40	1.07E-01	1.84E-01	6.18E-02	1.14E-01	6.69E-01	7.30E-01
PLR	Unscaled	155.38	181.35	5.50E-02	1.30E-01	2.53E-01	3.37E-01	**1.67E-03**	**1.00E-02**
NLR	Unscaled	2.38	2.81	2.00E-01	2.66E-01	1.03E-01	1.64E-01	**1.00E-02**	**3.27E-02**
MLR	Unscaled	0.32	0.43	**2.59E-03**	**1.91E-02**	**1.09E-02**	**3.27E-02**	8.10E-02	1.39E-01

Patients who developed liver metastases within 2 years after resection vs. metastasis-free patients. Evaluated with Mann–Whitney–Wilcoxon test (*) and verified with 2-way ANOVA accounting for PT location (#); significant variables (*p* < 0.05 and FDR < 0.1) are marked in bold

Next, we explored if the identified liver-metastasis-associated CBC parameters may predict liver-metastasis-free survival along with basic clinicopathological features (age at diagnosis, gender, PT location, pT and pN). Univariable Cox regression analysis selected seven variables associated with shorter time to liver metastasis development—male gender, pT, pN, RBC, HCT, LYMPH and MLR (Table 3). The final multivariable regression model narrowed to three features, pN, RBC (according to the lower margin of the normal range) and LYMPH (according to the middle of the normal range), as summarised in Table 3. Based on the survival analysis, we propose a simple liver metastasis risk score:

**Table 3:** Association of selected clinicopathologic features and CBC levels with liver-specific metastasis-free survival

Parameter		Univariable Cox regression				Multivariable Cox regression			
		HR	95% CI		*p*-value	HR	95% CI		*p*-value
Age at diagnosis	Continuous (unscaled)	1.03	0.99	1.06	1.66E-01	-	-	-	-
Gender	Male vs. female	2.55	1.10	5.91	**2.90E-02**	-	-	-	-
Primary tumour location	Left colon vs. right colon	1.47	0.56	3.86	4.38E-01	-	-	-	-
	Rectum vs. right colon	2.34	0.76	7.27	1.40E-01	-	-	-	-
pT	Continuous (unscaled)	1.95	0.99	3.82	**5.25E-02**	-	-	-	-
pN	Continuous (unscaled)	2.17	1.31	3.60	**2.64E-03**	2.12	1.28	3.52	**3.51E-03**
Red blood cells (RBC)	Continuous (scaled)	0.70	0.54	0.91	**8.09E-03**	-	-	-	-
	Below the normal range	2.91	1.33	6.37	**7.74E-03**	3.07	1.39	6.76	**5.42E-03**
Haematocrit (HCT)	Continuous (scaled)	0.73	0.55	0.99	**4.08E-02**	-	-	-	-
Lymphocytes (LYMPH)	Continuous (scaled)	0.43	0.21	0.87	**1.83E-02**	-	-	-	-
	Below the middle of the normal range	3.86	1.32	11.23	**1.34E-02**	3.11	1.06	9.15	**3.93E-02**
MLR	Continuous (unscaled)	5.69	1.46	22.08	**1.21E-02**	-	-	-	-

Patients who developed liver metastases within 2 years after resection vs. metastasis-free patients. Results of uni- and multivariable Cox regression analysis for the respective metastasis-free survival; significant variables (*p* < 0.1 in uni- and *p* < 0.05 in multivariable models) are marked in bold

$$\text{pN}\,\left(0/1/2\right)+1.5\,\left(\text{if RBC below the normal range}\right)+1.5\,\left(\text{if LYMPH below the middle of the normal range}\right)$$

In the next step, the obtained risk scores were subjected to ROC analysis (AUC = 0.79, 95% CI 0.70–0.89; Fig. 2g) to determine their ability to distinguish between patients with low and high risk of liver metastasis. Using a modified, specificity-focused, Youden approach (threshold with the maximal value of the sum of sensitivity + 2 × specificity), we established the cut-off at 2.75 (specificity = 0.83, sensitivity = 0.68). When applied to Cox regression analysis, the proposed risk categorisation yielded HR of 8.01 (95% CI 3.45–18.57, *p* < 0.001; C-index = 0.73; Fig. 2h) and an accuracy of 0.81. The most significant clinical predictor, pN, demonstrated lower predicting capacity, with the continuous increase of 1 (0/1/2) yielding ROC AUC of 0.65 (95% CI 0.54–0.77; Fig. 2g) and HR of 2.12 (95% CI 1.28–3.52, *p* < 0.001; Fig. 2i).

### Lung metastases

**Fig. 3:**
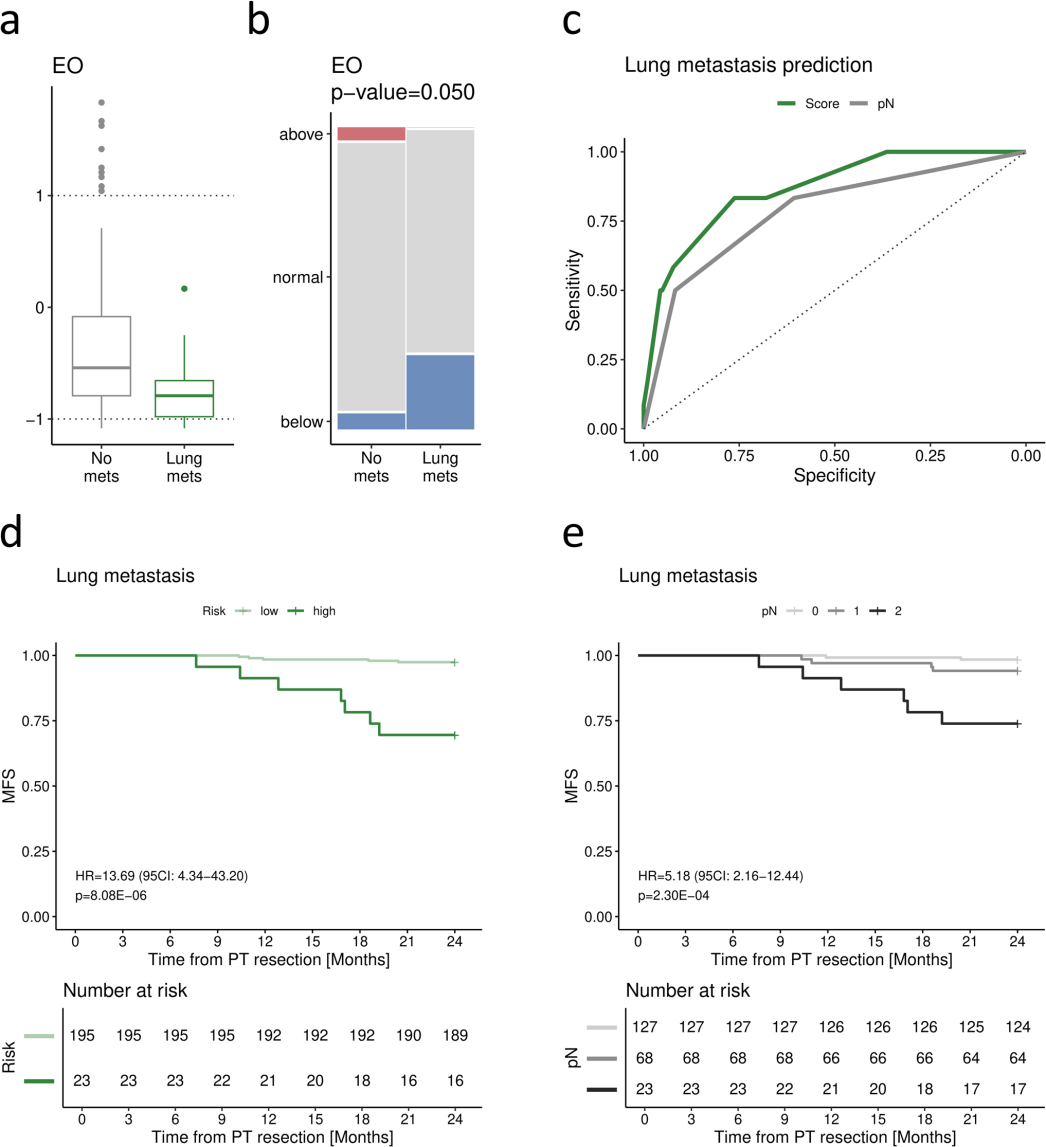
Features associated with lung metastasis. Distribution of EO (**a**, **b**) between patients who developed lung metastases (*n* = 12) vs. metastasis-free patients (*n* = 206). Continuous EO (**a**) is presented as scaled to the normal range. Categorisation performed based on the lower margin, middle and upper margin of the normal range (**b**). Evaluated with the Mann–Whitney–Wilcoxon test and verified with 2-way ANOVA accounting for PT location for continuous variables (*p*-values and FDR presented in Table 5), or Fisher’s exact test for categorical variables. Receiver operating characteristic (ROC) curves for predicting dissemination to the lungs based on the combined score (green) and pN only (grey) (**c**). Kaplan–Meier curves for lung metastasis-free survival (MFS) for the combined score dichotomised according to the cut-off value selected with the specificity-focused Youden approach (**d**) as well as for pN only (**e**). Hazard ratio (HR) and the corresponding 95% confidence interval (CI) estimated with Cox proportional hazard model; pN analysed for the effect of a continuous increase (0/1/2)

Similarly to the liver, within the first 2 years post-resection, lung metastases were most frequent in patients with stage III disease (10/91, 11%; *p* = 0.012; Table 4), especially with pN2 tumours (6/23, 26%; *p* < 0.001). In addition, the patients who developed metastases to the lungs presented lower preoperative levels of EO (Fig. 3a; Table 5), which was significant after reference-based categorisation (*p* = 0.050; Fig. 3b); thus, the counts were dichotomised according to the lower margin of the normal range for further analyses.

**Table 4:** Comparison of clinicopathological characteristics between patients developing early lung metastases and metastasis-free patients

Parameter		No mets (*n* = 206)		Lung mets (*n* = 12)		*p*-value
		*n*	%	*n*	%	
Age at diagnosis	Median (IQR)	66	59–73	71	68–74	9.70E-02
Gender	Female	116	56%	6	50%	7.68E-01
	Male	90	44%	6	50%	
Primary tumour location	Right colon	73	35%	1	8%	1.01E-01
	Left colon	103	50%	9	75%	
	Rectum	30	15%	2	17%	
pT	pT1	9	4%	0	0%	1.57E-01
	pT2	41	20%	0	0%	
	pT3	135	66%	9	75%	
	pT4	21	10%	3	25%	
pN	pN0	125	61%	2	17%	**1.62E-04**
	pN1	64	31%	4	33%	
	pN2	17	8%	6	50%	
Stage	I	42	20%	0	0%	**1.19E-02**
	II	83	40%	2	17%	
	III	81	39%	10	83%	

Patients who developed lung metastases within 2 years after resection vs. metastasis-free patients. Evaluated with Mann–Whitney–Wilcoxon test (Age at diagnosis) and with Fisher’s exact test (other variables); significant comparisons (*p* < 0.05) are marked in bold

**Table 5:** Comparison of complete blood counts between patients developing early lung metastases and metastasis-free patients

Parameter		No mets	Lung mets	Lung mets vs. no mets		Lung metastasis		Location	
		Median	Median	*p*-value*	FDR*	*p*-value^#^	FDR^#^	*p*-value^#^	FDR^#^
Red blood cells (RBC)	Scaled	−0.02	−0.08	9.92E-01	9.92E-01	8.91E-01	9.23E-01	**2.76E-02**	**9.46E-02**
Haemoglobin (HGB)	Scaled	−0.80	0.08	6.91E-02	4.15E-01	1.05E-01	2.09E-01	**1.86E-08**	**4.47E-07**
Haematocrit (HCT)	Scaled	−0.78	−0.28	2.31E-01	7.34E-01	2.85E-01	4.60E-01	**9.15E-08**	**1.10E-06**
Platelets (PLT)	Scaled	0.03	0.10	7.42E-01	8.09E-01	9.23E-01	9.23E-01	**2.58E-05**	**2.07E-04**
Neutrophils (NEUTR)	Scaled	0.02	0.12	4.95E-01	7.50E-01	2.41E-01	4.46E-01	**6.58E-03**	**3.16E-02**
Lymphocytes (LYMPH)	Scaled	−0.11	−0.16	5.63E-01	7.50E-01	3.96E-01	5.27E-01	3.40E-01	4.79E-01
Monocytes (MONO)	Scaled	−0.01	0.23	2.65E-01	7.34E-01	7.56E-01	8.64E-01	3.07E-01	4.60E-01
Eosinophils (EO)	Scaled	−0.54	−0.79	**2.00E-02**	2.39E-01	**4.50E-02**	1.35E-01	1.02E-01	2.09E-01
Basophils (BASO)	Scaled	−0.40	−0.40	4.19E-01	7.50E-01	8.06E-01	8.79E-01	5.26E-01	6.32E-01
PLR	Unscaled	155.38	162.94	6.46E-01	7.75E-01	4.64E-01	5.86E-01	**2.29E-03**	**1.38E-02**
NLR	Unscaled	2.38	2.71	5.48E-01	7.50E-01	7.06E-02	1.88E-01	**1.22E-02**	**4.86E-02**
MLR	Unscaled	0.32	0.36	3.06E-01	7.34E-01	3.06E-01	4.60E-01	8.05E-02	1.93E-01

Patients who developed lung metastases within 2 years after resection vs. metastasis-free patients. Evaluated with Mann–Whitney–Wilcoxon test (*) and verified with 2-way ANOVA accounting for PT location (#); significant variables (*p* < 0.05 and FDR < 0.1) are marked in bold

When analysed with clinicopathological characteristics in univariable Cox regression, EO predicted lung-metastasis-free survival along with tumour location, pT and pN (Table 6). The final multivariable model included three features—location, pN and EO (below the lower margin of the normal range; Table 6). Accordingly, we propose a simple risk score of lung metastasis development:

**Table 6:** Association of selected clinicopathologic features and CBC levels with lung-specific metastasis-free survival

Parameter		Univariable Cox regression				Multivariable Cox regression			
		HR	95% CI		*p*-value	HR	95% CI		*p*-value
Age at diagnosis	Continuous (unscaled)	1.04	0.99	1.10	1.52E-01	-	-	-	-
Gender	Male vs. female	1.27	0.41	3.95	6.76E-01	-	-	-	-
Primary tumour location	Left colon vs. right colon	6.06	0.77	47.82	**8.75E-02**	6.59	0.83	52.40	**7.46E-02**
	Rectum vs. right colon	4.79	0.43	52.79	2.01E-01	2.84	0.25	32.33	4.01E-01
pT	Continuous (unscaled)	3.18	1.18	8.56	**2.22E-02**	-	-	-	-
pN	Continuous (unscaled)	4.49	2.06	9.78	**1.56E-04**	5.18	2.16	12.44	**2.30E-04**
Eosinophils (EO)	Continuous (scaled)	0.18	0.03	1.03	**5.44E-02**	-	-	-	-
	Below the normal range	5.34	1.45	19.75	**1.20E-02**	7.39	1.87	29.18	**4.32E-03**

Patients who developed lung metastases within 2 years after resection vs. metastasis-free patients. Results of uni- and multivariable Cox regression analysis for the respective metastasis-free survival; significant variables (*p* < 0.1 in uni- and *p* < 0.05 in multivariable models) are marked in bold

$$2\,\left(\text{if PT in left colon}\right)+1\,\left(\text{if PT in rectum}\right)+1.5\times \text{pN}\,\left(0/1/2\right)+2\,\left(\text{if EO below the normal range}\right)$$

In the next step, ROC analysis demonstrated its high efficiency (AUC = 0.87, 95% CI 0.77–0.97; Fig. 3c), while the specificity-focused Youden approach established the cut-off at 3.75 (specificity = 0.92, sensitivity = 0.58). Cox regression proved the substantial predictive capacity of the proposed categorisation, yielding HR of 13.69 (95% CI 4.34–43.20, *p* < 0.001; C-index = 0.75; Fig. 3d) and an accuracy of 0.90. Again, compared to the proposed model, pN presented lower efficiency with the continuous increase of 1 (0/1/2) yielding ROC AUC of 0.78 (95% CI 0.64–0.92; Fig. 3c) and HR of 5.18 (95% CI 2.16–12.44, *p* < 0.001; Fig. 3e).

## Discussion

Here, we aimed to identify routine blood test parameters associated with subclinical metastases or premetastatic niche formation. Lung and liver metastases are consistently reported to have specific biological (particularly immunological) features that differ both from each other and other metastatic sites [6, 28–31]. The role of the systemic immune response in metastasis formation is evident [39] and seems organ-specific [6]. Moreover, the overall immunologic condition may be to some extent reflected by peripheral blood composition. A machine learning-based algorithm (named ColonFlag), relying on CBC, age and gender was proposed to detect early colorectal cancer [40, 41]. We hypothesised that the process of premetastatic niche formation might impact CBC parameters with differences depending on the targeted site. For this purpose, we employed a thoroughly and comprehensively curated dataset, assuring adequate staging in all cases and excluding those with underdiagnosed overt metastases. Additionally, we focused only on early CRC metastases (within 2 years after resection), assuming that with increasing time to progression, the putative CBC changes are less manifested in preoperative blood samples. To our knowledge, this is the first study attempting to identify CRC patients at higher risk of developing site-specific metastasis based on the preoperative CBC parameters in combination with clinicopathological characteristics.

To date, organ-specific metastasis prediction in CRC has primarily relied on clinicopathological and radiological data. The former mainly originated from the Surveillance, Epidemiology and End Results (SEER) database [42], with numerous models and nomograms proposed for predicting dissemination to the liver [43], lungs [44–47] or multiple organs [48–50]. In turn, radiological approaches predominantly exploit machine learning-assisted radiomics models that predict metachronous CRC metastases both in a general [51] and an organ-specific manner, e.g. to the liver [52–56] or lungs [57].

Organotropism plays a pivotal role in CRC metastasis, making it essential to understand the underlying mechanisms for both diagnostic and therapeutic purposes [6, 58]. Although the liver is always the most common target of CRC metastatic spread, the specific frequencies of the involvement of each organ are well known to depend on the location of the primary tumour within the large intestine. In turn, the location also significantly affects peripheral blood parameters, including RBC [59], haemoglobin [59], haematocrit [59], lymphocytes [59, 60], platelets [60], PLR [60, 61], NLR [60] and MLR [60]. Therefore, in the first step, we excluded the parameters whose association with liver and lung metastases was solely a reflection of their association with tumour location.

In our CRC cohort, the magnitude of lymph node involvement (pN stage) emerged as a common factor linked to both liver and lung metastases. However, distinct blood count profiles differentiated these sites. Liver metastasis risk was associated with erythrocytopenia and lymphopenia (below 2000 lymphocytes per microliter), while eosinopenia characterised patients at risk of lung metastasis. These CBC alterations may serve as early markers of liver and lung dysfunction during the initial stages of metastatic foci formation, which remain clinically undetectable.

Liver metastasis in CRC has not been directly linked to erythrocytopenia before, though Sala et al. observed reduced erythrocyte-related parameters (HGB, RBC, MCV, MCH and MCHC) months prior to liver metastasis diagnosis, implicating anaemia as a marker of liver dissemination [62]. Erythrocytopenia may indicate altered liver function, systemic inflammation driving hepcidin-mediated iron sequestration, or tumour-induced anaemia. Measures of red blood cell anisocytosis, such as RDW and RDW-CV, have also been associated with poor survival and liver dissemination after CRC resection [63–66], reflecting systemic changes in erythropoiesis that may favour metastasis. Similarly, preoperative lymphopenia emerged as a potential contributor to liver metastasis risk in our study, likely reflecting systemic immunosuppression or disrupted immune surveillance. Cheng et al. reported elevated SII (Systemic Inflammatory Index, platelet count × NLR) in patients with metachronous liver metastases [66], while inflammation-related markers incorporating lymphocyte counts, such as PLR, NLR, MLR and SIS, have been linked to CRC prognosis [67–74].

Lung metastasis risk, in contrast, was associated with primary tumour location and eosinopenia. Eosinophils may play a protective role against lung metastases, supported by reports linking eosinophil levels to CRC outcomes [75]. Both eosinopenia [76] and eosinophilia [59] have been associated with shorter DFS. Eosinopenia in lung metastasis may result from systemic immune suppression, tumour-induced eosinophil sequestration or cytokine-mediated inhibition of eosinophil production, all of which warrant further study.

Several limitations of our work have to be acknowledged. Firstly, it is a single-centre retrospective study based on a relatively homogeneous Caucasian population, which limits the generalisation of the results. On the other hand, thanks to the thorough manual curation of medical records, including radiological data, we could exclude underdiagnosed overt metastases at the time of primary tumour resection. Yet, it resulted in a relatively low number of events, lung metastases in particular, and decreased the statistical power of the analysis. Secondly, some clinicopathological data was missing or incomplete for a significant proportion of the patients, including information on the adjuvant treatment applied, venous and lymphovascular invasion or preoperative levels of the carcinoembryonic antigen (CEA), a marker used to monitor CRC progression [77, 78]. Finally, ctDNA emerges as a valuable prognostic biomarker, which was documented in the DYNAMIC trial [19]. The SCRUM-Japan GOZILA study also indicated notable variations in ctDNA levels among CRC patients with metastases to different organs, with pulmonary and peritoneal dissemination manifesting with lower ctDNA levels [79]. On the other hand, the availability of such methods is limited, in contrast to the simple CBC parameters, which, when validated, may be easily implemented also in developing countries.

In summary, our findings indicate that early CRC metastases to the liver and lungs are associated with partially divergent clinicopathological and peripheral blood features. Additionally, we propose simple, clinically implementable scores, based on routinely assessed parameters, which may identify patients with an increased risk of developing liver and lung metastases. Calculating these risk scores at the time of primary resection could help stratify patients for targeted surveillance of specific organs, enabling early detection of metastases, or even for adjuvant treatment. After validation in independent cohorts along with gold-standard methods for monitoring CRC progression, such as CEA and ctDNA, these scores may provide easily available prognostic information. Finally, the need for translational research focused on the underlying biological differences between CRC metastasis development in different organs has to be emphasised.

## Supplementary Information

Below is the link to the electronic supplementary material.

Supplementary file1 (PDF 245 KB)

## Data Availability

Dataset analysed in this study is available from the corresponding author upon reasonable request.
