# Supplementary material for: Complete blood counts as potential risk factors of early dissemination to liver and lungs in resected colorectal cancer: a retrospective cohort study
Source: Int J Colorectal Dis. 2025 Jan 21;40(1):21. doi: 10.1007/s00384-024-04802-9 (PMC11750913; doi:10.1007/s00384-024-04802-9)
Supplement: Supplementary file 1 — Supplementary file1 (PDF 245 KB) [file 384_2024_4802_MOESM1_ESM.pdf]

Complete blood counts as potential risk factors of early dissemination to liver and lungs in resected colorectal cancer: a retrospective cohort study

Marta Popęda [1#], Jolanta Żok [2], Bartłomiej Tomasik [3], Renata Duchnowska [2], Michał Bieńkowski [1]

1 Medical University of Gdańsk, Department of Pathomorphology, Gdańsk, Poland

2 Military Institute of Medicine, Department of Oncology, Warsaw, Poland

3 Medical University of Gdańsk, Department of Oncology and Radiotherapy, Gdańsk, Poland

[#] corresponding author: Marta Popęda, PhD; e-mail: marta.popeda@gumed.edu.pl

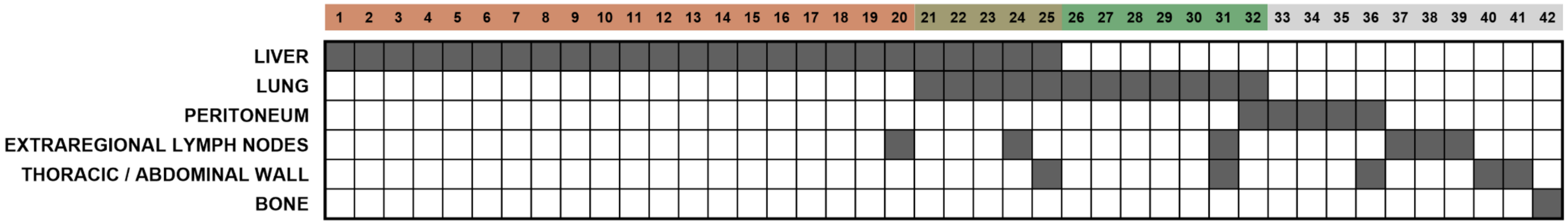

Figure S1. Detailed distribution of metastatic sites in patients who developed metastases within two years after primary CRC resection

Patients with liver but not lung metastases (n=20) are marked in orange, combined liver and lung metastases (n=5) in brown, lung but not liver metastases (n=7) in green, and metastases to other organs (n=10) in grey.

Table S1. Gender-specific normal range values for complete blood counts and the values used for scaling

Parameter		Reference			Scaling		
		Gender	Range	Unit	-1	0	1
Red blood cells	RBC	Females	3.8-4.8	x10\^12/l	3.8	4.3	4.8
		Males	4.5-5.5	x10\^12/l	4.5	5	5.5
Haemoglobin	HGB	Females	12-15	g/dl	12	13.5	15
		Males	13-17	g/dl	13	15	17
Haematocrit	HCT	Females	36-45	%	36	40.5	45
		Males	40-50	%	40	45	50
Platelets	PLT	Universal	150-410	x10\^9/l	150	280	410
Neutrophils	NEUTR	Universal	2-7	x10\^9/l	2	4.5	7
Lymphocytes	LYMPH	Universal	1-3	x10\^9/l	1	2	3
Monocytes	MONO	Universal	0.2-1	x10\^9/l	0.2	0.6	1
Eosinophils	EO	Universal	0.02-0.5	x10\^9/l	0.02	0.26	0.5
Basophils	BASO	Universal	0-0.1	x10\^9/l	0	0.05	0.1

Table S2. Comparison of clinicopathological characteristics between mucinous and tubular adenocarcinomas

Evaluated with Mann-Whitney-Wilcoxon test (Age at diagnosis) and with Fisher's exact test (other variables); significant comparisons (p<0.05) are marked in bold.

Parameter		Mucinous (n=20)		Tubular (n=248)		p-value
		n	%	n	%	
Age at diagnosis	Median (IQR)	76	69-81	67	60-74	**1.65E-03**
Gender	Female	10	50%	135	54%	8.17E-01
	Male	10	50%	113	46%	
Primary tumour location	Right colon	15	75%	85	34%	**1.15E-03**
	Left colon	5	25%	124	50%	
	Rectum	0	0%	39	16%	
pT	pT1	0	0%	9	4%	4.24E-01
	pT2	1	5%	45	18%	
	pT3	16	80%	162	65%	
	pT4	3	15%	32	13%	
pN	pN0	14	67%	143	58%	6.54E-01
	pN1	5	24%	78	31%	
	pN2	2	10%	27	11%	
Stage	I	1	5%	44	18%	9.13E-02
	II	13	65%	99	40%	
	III	6	30%	105	42%	
Distant metastasis	No	19	95%	206	83%	1.00E+00
	Liver	1	5%	20	8%	
	Lung	0	0%	7	3%	
	Concurrent liver and lung	0	0%	5	2%	
	Other organs	0	0%	10	4%	

Table S3. Comparison of complete blood counts between mucinous and tubular adenocarcinomas

Evaluated with Mann-Whitney-Wilcoxon test (*) and verified with 2-way ANOVA accounting for PT location (#); significant variables ($p<0.05$ and FDR<0.1) are marked in bold.

Parameter		Tubular	Mucinous	Mucinous vs Tubular		Histology		Location	
		median	median	p-value *	FDR *	p-value #	FDR #	p-value #	FDR #
Red blood cells (RBC)	Scaled	-0.09	-0.57	**1.05E-02**	**3.16E-02**	**4.37E-03**	**1.31E-02**	**9.51E-03**	**2.49E-02**
Haemoglobin (HGB)	Scaled	-0.86	-2.67	**3.62E-04**	**4.35E-03**	**5.17E-06**	**3.10E-05**	**1.57E-10**	**3.77E-09**
Haematocrit (HCT)	Scaled	-0.82	-2.26	**9.20E-04**	**5.52E-03**	**1.52E-05**	**7.30E-05**	**2.29E-09**	**2.74E-08**
Platelets (PLT)	Scaled	0.04	0.64	**2.27E-03**	**9.06E-03**	**4.38E-04**	**1.75E-03**	**2.65E-06**	**2.12E-05**
Neutrophils (NEUTR)	Scaled	0.03	0.31	1.10E-01	1.80E-01	5.38E-02	**9.93E-02**	**1.04E-02**	**2.49E-02**
Lymphocytes (LYMPH)	Scaled	-0.15	-0.11	4.73E-01	4.73E-01	7.19E-01	7.51E-01	7.07E-01	7.51E-01
Monocytes (MONO)	Scaled	0.03	0.10	9.96E-02	1.80E-01	1.35E-01	2.03E-01	1.27E-01	2.03E-01
Eosinophils (EO)	Scaled	-0.58	-0.35	1.20E-01	1.80E-01	1.73E-01	2.31E-01	1.54E-01	2.18E-01
Basophils (BASO)	Scaled	-0.40	-0.10	1.64E-01	2.18E-01	**4.47E-02**	**8.94E-02**	4.27E-01	5.40E-01
PLR	Unscaled	157.58	189.81	**4.01E-02**	**9.62E-02**	6.21E-01	7.10E-01	**1.24E-03**	**4.24E-03**
NLR	Unscaled	2.40	2.59	4.22E-01	4.73E-01	4.98E-01	5.98E-01	**2.33E-02**	**5.09E-02**
MLR	Unscaled	0.33	0.34	4.57E-01	4.73E-01	7.56E-01	7.56E-01	6.16E-02	1.06E-01
